# Supplementary material for: Supra-operonic clusters of functionally related genes (SOCs) are a source of horizontal gene co-transfers
Source: Sci Rep. 2017 Jan 9;7:40294. doi: 10.1038/srep40294 (PMC5220362; doi:10.1038/srep40294)
Supplement: Supplementary Figures [file srep40294-s1.pdf]

Supra-operonic clusters of functionally related genes (SOCs) are a source of horizontal gene co-transfers

Tin Yau Pang[1,*] and Martin J. Lercher[1]

[1] Institute for Computer Science, Heinrich Heine University, Düsseldorf, 40225, Germany

* Corresponding author
Email: pang@hhu.de

SUPPLEMENTARY FIGURES

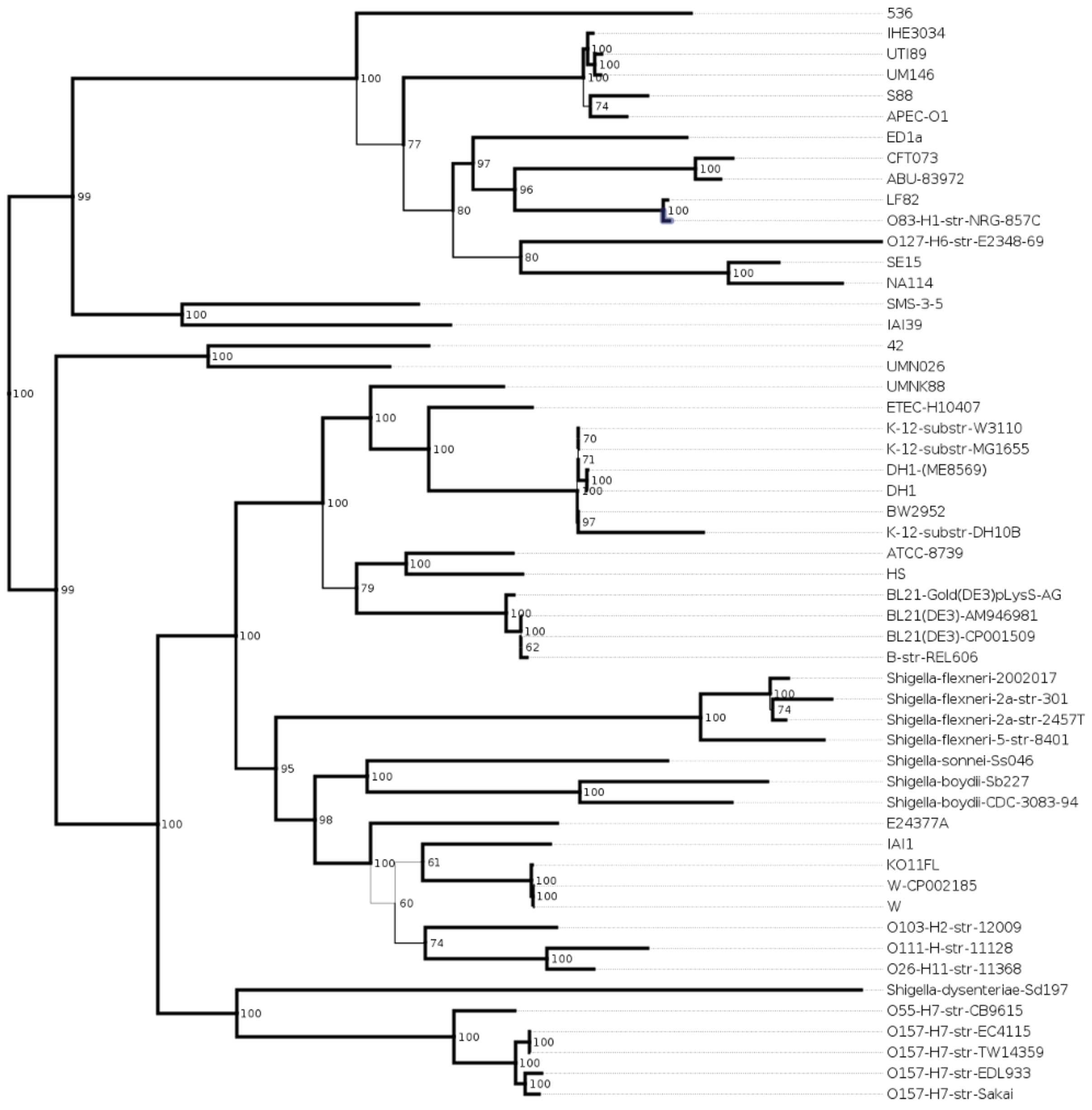

Supplementary Fig. S1. Phylogenetic tree of the 53 strains considered. Phylogenetic tree representing vertical inheritance among the 53 *E. coli* and *Shigella* strains. The thickness of each branch and the number to its right correspond to its bootstrap value.

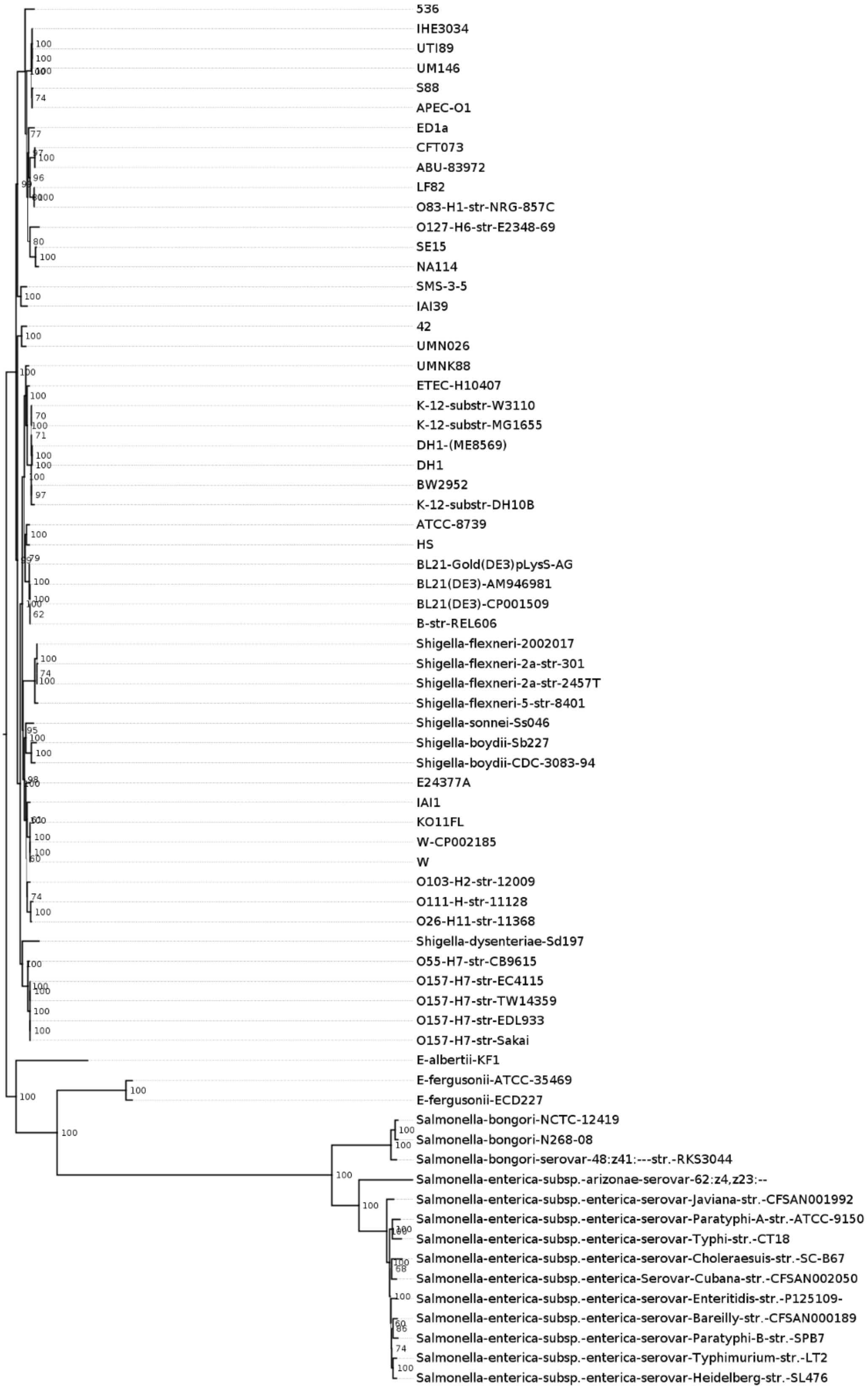

Supplementary Fig. S2. **Phylogenetic tree of the 53 considered and 17 outgroup strains.** Phylogenetic tree of the 53 *E. coli* and *Shigella* strains, plus another 17 outgroup strains. The thickness of each branch and the number to its right correspond to its bootstrap value.

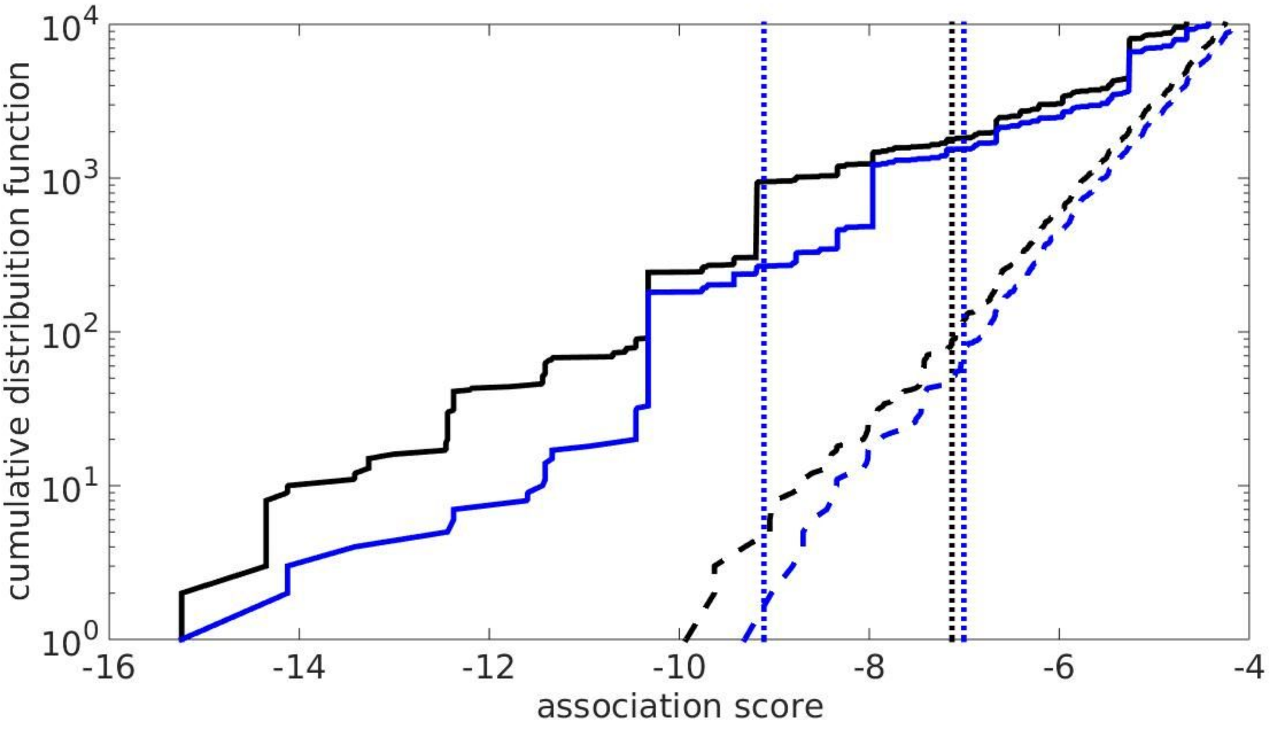

Supplementary Fig. S3. Cumulative distribution function of pairwise gene association scores. Black lines: reproduction of Fig. 1, distribution of co-gain score calculated with an inclusive set of gained-genes; blue lines: distribution of co-gain score calculated with a stringent set of gained-genes. Many gene-pairs show much stronger co-gain scores (solid lines) than expected from random HGT (dashed lines). Distribution of the score for pairwise gene associations *t* in the empirical data for the inclusive set of gained-genes (black solid line) and for the stringent set of gained-genes (blue solid line), and the null model calculated with the inclusive set of gained-genes (black dashed line) and with the stringent set of gained-genes (blue dashed line), based on the maximum-likelihood ancestral genome reconstructions. The two black vertical dotted lines at *t*=-7.1289 and -9.1090 correspond to FDRs of 0.05 and 0.005 for the inclusive set of gained-genes, respectively; the two blue vertical dotted lines at *t*=-7.0039 and -9.1090 correspond to FDRs of 0.05 and 0.005 for the stringent set of gained-genes, respectively.

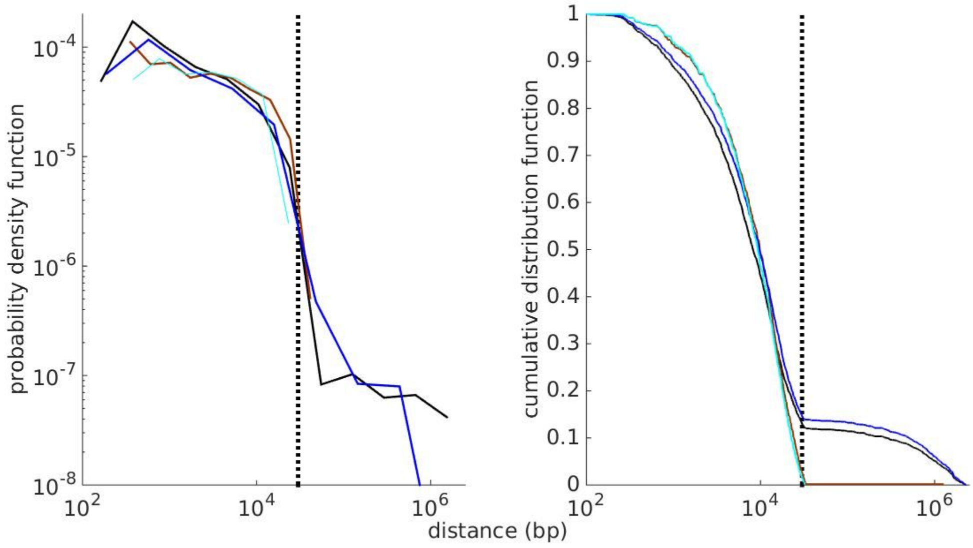

Supplementary Fig. S4. Cumulative and probability distribution of the minimal distances of the cogain gene pairs for the inclusive HGT set and high-confidence HGT set. Probability density (left) and cumulative distribution function of genomic distances between co-gained gene pairs in the inclusive HGT set (black), pairs with both genes non-phage-associated in the inclusive set (brown), co-gained gene pairs the high confidence set (blue), and pairs with both genes non-phage-associated in the high confidence set (cyan), at FDR 0.05. The vertical dotted line indicates a genomic distance of 30kb.

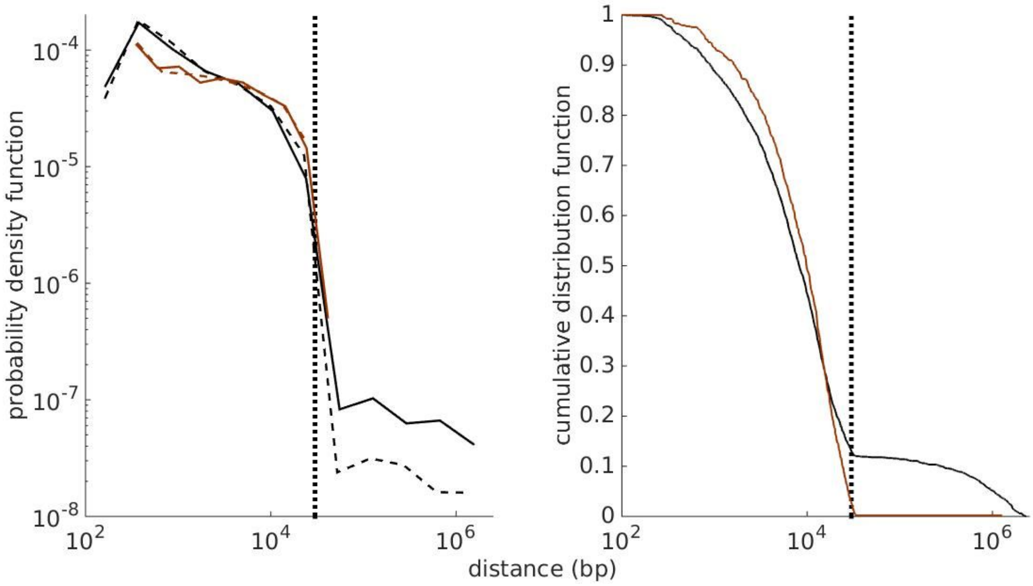

Supplementary Fig. S5. Cumulative and probability distribution of the minimal distances of the cogain gene pairs. Probability density (left) and cumulative distribution function (right, reproduction of Fig. 2) of genomic distances between co-gained gene pairs at FDR 0.05 (black solid curve) and 0.005 (black dashed curve), and also pairs at FDR 0.05 with both genes non-phage-associated (FDR 0.05: brown solid curve; FDR 0.005: brown dashed curve). The vertical dotted line indicates a genomic distance of 30kb.

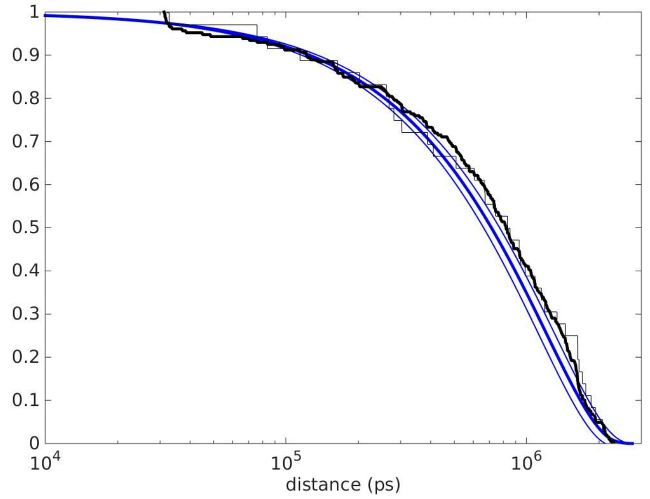

Supplementary Fig. S6. Cumulative distance distribution of random and cogain gene pairs. Comparison of cumulative distribution of distance between random gene pairs on a genome, averaged over five *E. coli* strains (BL21(DE3)-AM946981, O157-H7-str-Sakai, APEC-O1, IAI1 and K-12 MG1655; thick blue curve for mean, and the two thin blue curves above and below the mean curve for one standard deviation), with the rescaled cumulative distribution function of genomic distances between co-gained gene pairs at FDR 0.05 (thick black curve, rescaled by a factor 7.87) and 0.005 (thin black curve, rescaled by a factor 26.3).

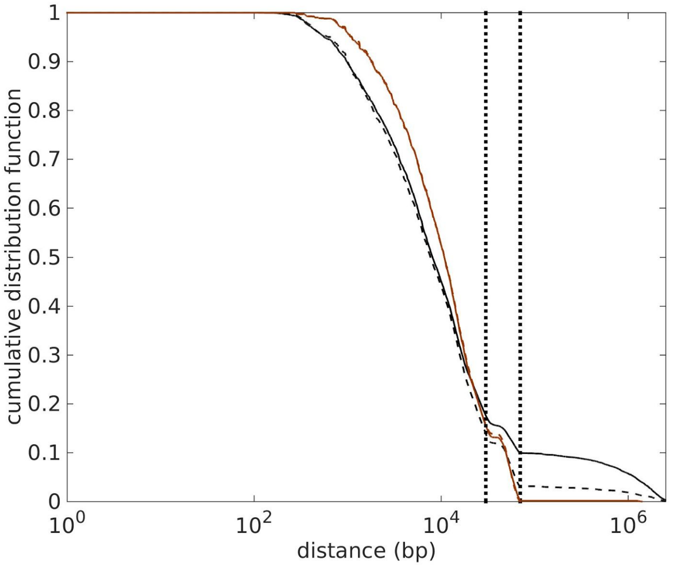

Supplementary Fig. S7. **Cumulative distance distribution of all the cogain allele pairs.** Cumulative distribution function of the genomic distances between all co-gained gene pairs (black) and between non-phage-associated gene pairs (brown), at FDR 0.05 (solid curves) and 0.005 (dashed curves), weighing every occurrence of a gene pair across different extant genomes; the two dotted vertical lines correspond to 30kb and 70kb. The features of the four curves largely conserved with the distribution that weight each gene pairs equally (Fig. 2 and Supplementary Fig. S5), except that there is one more kink followed by a long tail at 70kb for the curves of all co-gained pairs (black curves) but no tail for the non-phage-associated gene pairs (brown curves).

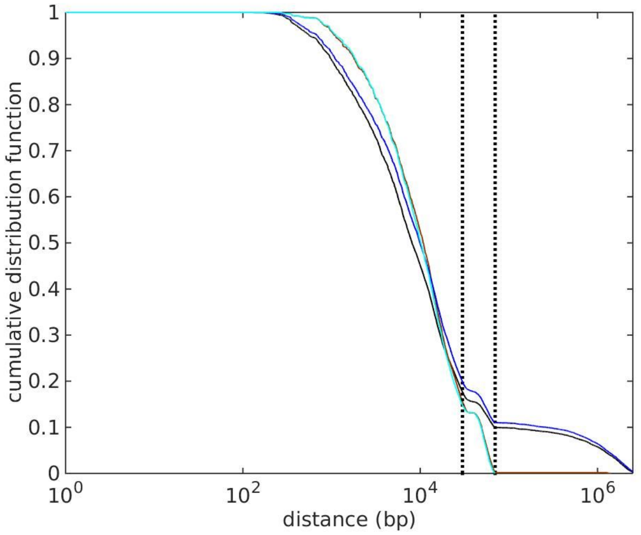

Supplementary Fig. S8. **Cumulative distance distribution of co-gain allele pairs of the inclusive and high-confidence HGT set.** Cumulative distribution function of the genomic distances between all co-gained gene pairs of the inclusive HGT set (black), between non-phage-associated co-gained gene pairs of the inclusive HGT set (brown), between all co-gained gene pairs in the high-confidence set (blue) and between non-phage-associated co-gained gene pairs of the high-confidence set (cyan), at FDR 0.05, weighting every occurrence of a gene pair across different extant genomes the same; the two dotted vertical lines correspond to 30kb and 70kb. The features of the four curves largely conserved with the distribution that weight each gene pairs equally (Fig. 2 and Supplementary Fig. S5), except that there is one more kink followed by a long tail at 70kb for the curves of all co-gained pairs (black and blue curves) but no tail for the non-phage-associated gene pairs (brown and cyan curves).

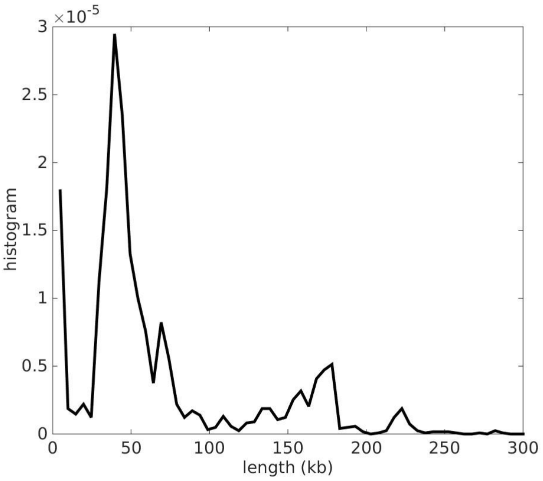

Supplementary Fig. S9. Size distribution of phage DNA sequences on the EMBL database

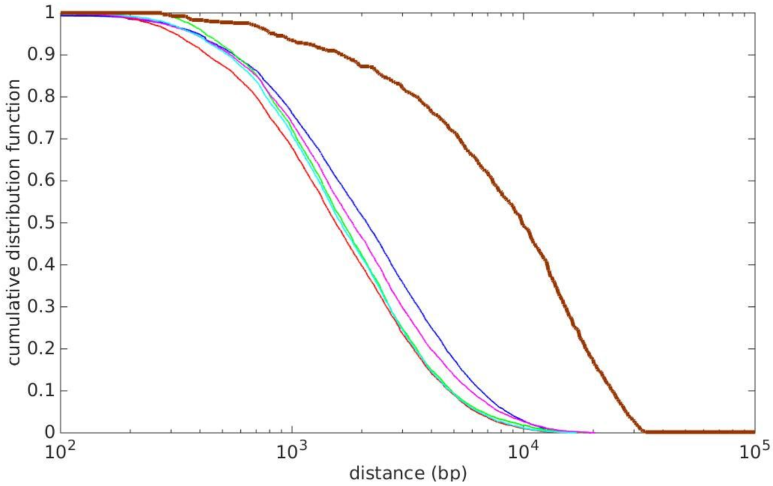

Supplementary Fig. S10. Cumulative distance distribution of cogain gene pairs and gene pairs within operons. Cumulative distribution function of the genomic distances between non-phage-associated co-gained orthologous gene family pairs at FDR 0.05 (thick brown curves, respectively), and also between gene pairs within operons of BL21(DE3)-AM946981 (blue), O157-H7-str-Sakai (red), APEC-O1 (green), IAI1 (magenta) and K-12 MG1655 (cyan).

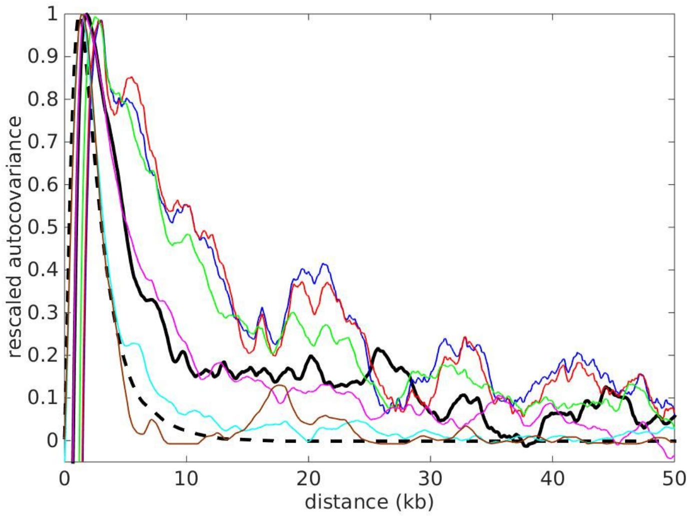

Supplementary Fig. S11. **Rescaled gene co-occurrence autocovariance.** Rescaled CO-AC of genes, averaged over five *E. coli* strains (BL21(DE3)-AM946981, O157-H7-str-Sakai, APEC-O1, IAI1 and K-12 MG1655), at five different MI cutoffs: 0.0001 (blue), 0.001 (red), 0.01 (green), 0.1 (magenta), 0.5 (cyan) and 0.8 (brown); as well as rescaled GO-AC of genes (black solid), and GO-AC calculated only with operons in *E. coli* K-12 MG1655 (black dashed). At small MI cutoff, an increase in cutoff value does not change the trend of the AC curve; when the MI cutoff is beyond 0.01, an increase in MI cutoff value leads to a faster decay of the AC curve. Further, the noisy fluctuation of AC causes a drop below zero for the cutoff=0.001 curve (red) at 15kb, even though the trend of the curve decays to the background at a much larger distance; this distorts the approximation of pairwise distance distribution of genes in subsequent analysis and leads to poorer uber-operon mode filt.

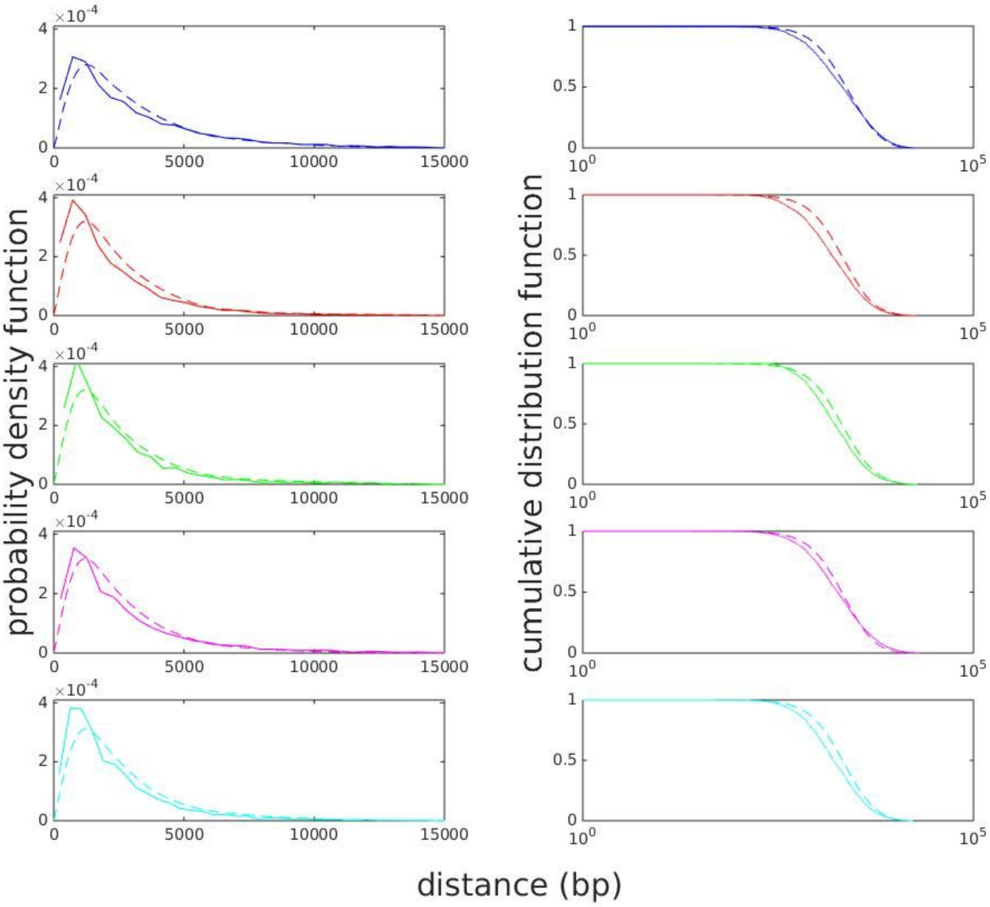

Supplementary Fig. S12. Real and estimated distance distribution of gene pairs within operons on five representative *E. coli* genomes. Probability density (left) and cumulative distribution (right) function of distances of gene pairs in operons of *E. coli* BL21(DE3)-AM946981 (blue solid), O157-H7-str-Sakai (red solid), APEC-O1 (green solid), IAI1 (magenta solid) and K-12 MG1655 (cyan solid), and their approximations estimated from the normalized and rescaled GO-AC (n=1) of nucleotides within operons (dashed curves).

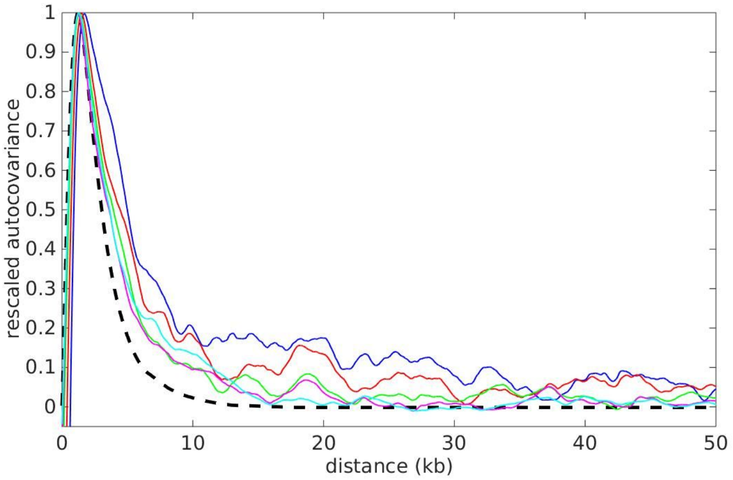

Supplementary Fig. S13. **Rescaled gene co-functioning auto-covariance of all gene pairs at different GO cutoffs.** Rescaled GO-AC of genes, averaged over five *E. coii* strains (BL21(DE3)-AM946981, O157-H7-str-Sakai, APEC-O1, IAI1 and K-12 MG1655), where gene pairs with at least 1 (blue), 2 (red), 3 (green), 4 (magenta) or 5 (cyan) common GO terms to be considered co-functioning. Increase in GO term cutoff leads to faster decay to background level, gradually approaching the autocovariance curve of GO-AC of genes (cutoff at 1 common GO term) restricted within operons.

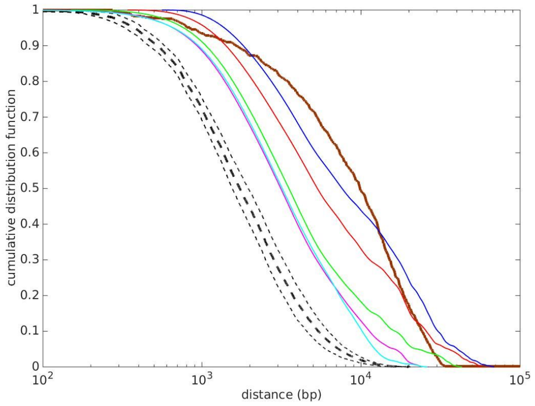

Supplementary Fig. S14. **Cumulative distance distribution of cogain gene pairs, gene pairs within operons and gene pairs in co-functioning clusters (estimated with various cutoffs).** Cumulative distribution of pairwise distance of genes in clusters estimated from GO-AC, with gene pairs having at least 1 (blue, *n*=1), 2 (red, *n*=2), 3 (green, *n*=3), 4 (magenta, *n*=4), or 5 (cyan, *n*=5) common GO terms to be considered co-functioning, averaged over five *E. coli* strains (BL21(DE3)-AM946981, O157-H7-str-Sakai, APEC-O1, IAI1 and K-12 MG1655); phage-associated genes are ignored; genes that have overlap with mobile elements are also ignored. Cumulative distribution of pairwise distance of non-phage-associated co-gained orthology gene family pairs (thick brown) as well as cumulative distribution of pairwise distance of genes in operons (averaged over the distribution curves of five different *E. coli* strains; thick dashed black curve for the mean, the two thin dashed black curves above and below the mean curve represents the one standard deviation boundary) for comparison.

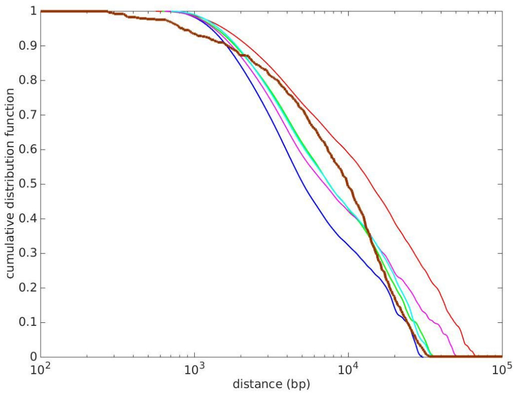

Supplementary Fig. S15. **Cumulative distance distribution of cogain gene pairs and gene pairs in co-functioning clusters (estimated with cutoff *n*=1).** Cumulative distribution of pairwise distance of genes in uber-operon, estimated using GO-AC that assumes each gene pair with at least one common GO term (*n*=1) to be functionally connected; the distribution is calculated based on the genes in five different *E. coli* strain: BL21(DE3)-AM946981 (blue solid), O157-H7-str-Sakai (red solid), APEC-O1 (green solid), IAI1 (magenta solid) and K-12 MG1655 (cyan solid); phage-associated genes are ignored; genes that have overlap with mobile elements are also ignored. The cumulative distribution of pairwise distance of non-phage-associated co-gained gene family pairs (thick brown) is displayed for comparison.

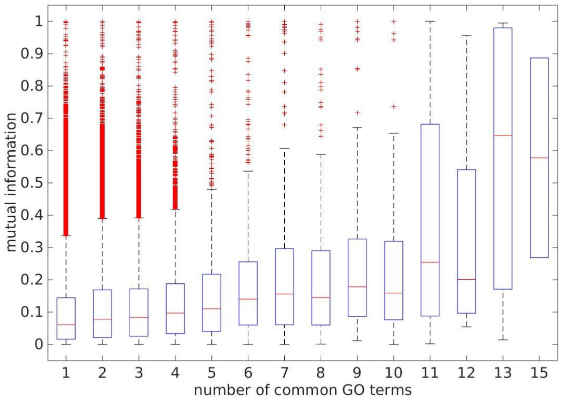

Supplementary Fig. S16. Box plot of mutual information vs number of common GO terms of the gene pairs. Box plot showing the MI distribution of orthologous gene family pairs that have defined MI value and assigned GO terms, categorized at different *n*>0, with *n* being the minimum number of common GO terms they share. Statistical analysis supports a positive correlation between MI and *n* (Spearman; ρ=0.0760, p<2.2E-16).

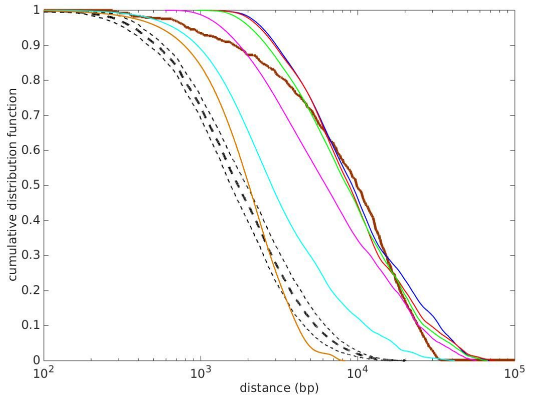

Supplementary Fig. S17. Cumulative distance distribution of cogain gene pairs, gene pairs within operons and gene pairs in co-occurrence cluster estimated from 233 γ-proteobacterial genomes. Cumulative distribution of pairwise distance of genes in uber-operon, estimated using CO-AC at MI cutoffs 0.0001 (blue), 0.001 (red), 0.01 (green), 0.1 (magenta), 0.5 (cyan) and 0.8 (orange), cumulative distribution of non-phage-associated co-gained gene family pairs (FDR 0.05, thick brown) and also cumulative distribution of pairwise distance of genes in operons averaged of five *E. coli* strains: BL21(DE3)-AM946981, O157-H7-str-Sakai, APEC-O1, IAI1 and K-12 MG1655 (thick dashed black curve for the mean of the five distributions, the two thin dashed black curves above and below the mean curve represent the boundary of one standard deviation). MI is calculated from the presence and absence profile of genes across 233 γ-proteobacterial species.

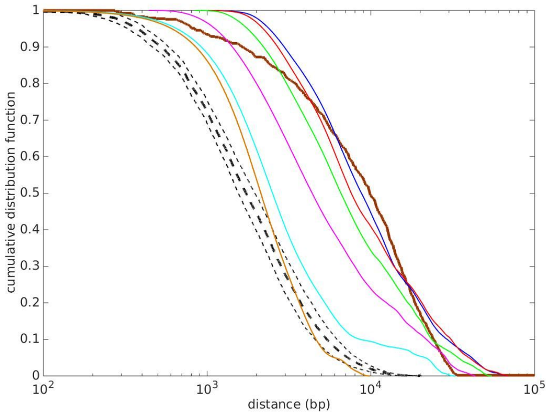

Supplementary Fig. S18. **Cumulative distance distribution of cogain gene pairs, gene pairs within operons and gene pairs in co-occurrence cluster estimated from 198 γ-proteobacterial genomes (excluding *E. coli* strains).** Cumulative distribution of pairwise distance of genes in uber-operon, estimated using CO-AC at MI cutoffs 0.0001 (blue), 0.001 (red), 0.01 (green), 0.1 (magenta), 0.5 (cyan) and 0.8 (orange), cumulative distribution of non-phage-associated co-gained gene family pairs (FDR 0.05, thick brown) and also cumulative distribution of pairwise distance of genes in operons averaged of five *E. coli* strains: BL21(DE3)-AM946981, O157-H7-str-Sakai, APEC-O1, IAI1 and K-12 MG1655 (thick dashed black curve for the mean of the five distributions, the two thin dashed black curves above and below the mean curve represent the boundary of one standard deviation). MI is calculated from the presence and absence profile of genes across 198 γ-proteobacterial species, which does not include any *E. coli* or *Shigella* genomes.

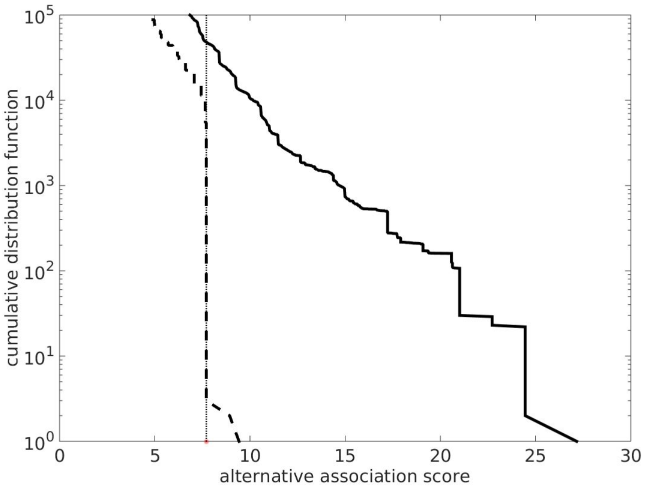

Supplementary Fig. S19. **Cumulative distribution function of alternative pairwise gene association score.** Distribution of the alternative score for pairwise gene associations *t* in the empirical data (solid line) and the null model (dashed line), which is analogous to Fig. 1 that makes use of the original association score. The vertical dotted line at *t*=-7.707 corresponds to both FDRs of 0.05 and 0.005.

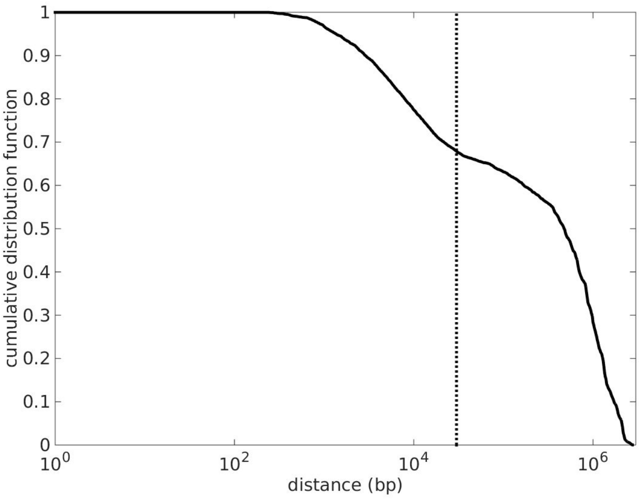

Supplementary Fig. S20. **Cumulative distance distribution function of cogain gene pairs inferred from the alternative pairwise gene association score.** The distance distribution of co-gained gene pairs predicted using the alternative association score. The kink at 30kb (dotted line) is still visible, but the long tails representing false detection is very strong and covers the majority of the distribution.

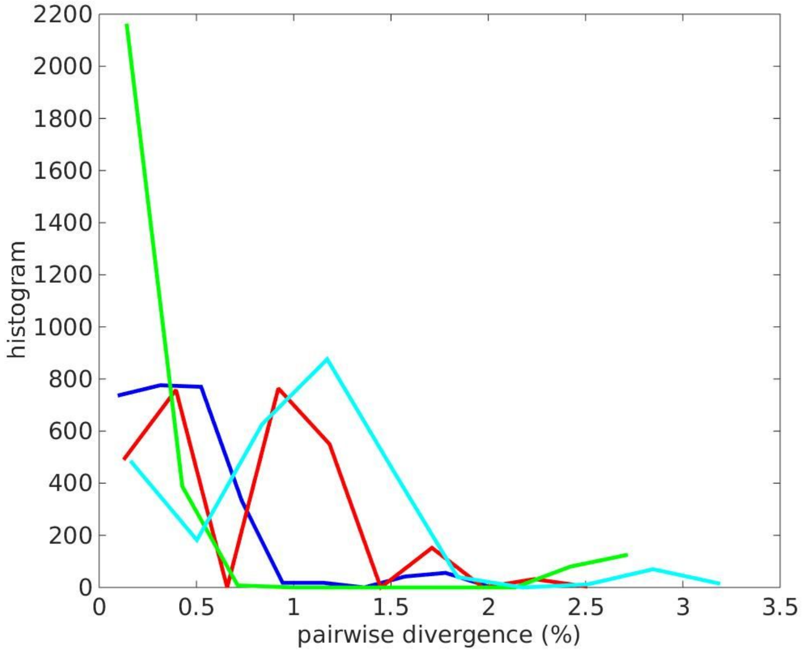

Supplementary Fig. S21. **Histogram of pairwise divergence of the methyl-directed mismatch repair (MMR) genes across 53 *E. coli* strains.** The four distribution curves correspond to mutS (blue), mutH (red), mutU / uvrD (green) and mutL (cyan).

SUPPLEMENTARY TABLES

Supplementary Table S1. List of the 53 E. coli strains and 17 outgroup strains used in the analysis; in addition there are 2 extra strains that were included in the determination of orthologous gene families with ProteinORTHO.
Supplementary Table S2. Output of ProteinORTHO performed on the 70 genomes in our study, plus the two additional E. coli strains SE11 and 55989, with orphan genes omitted.
Supplementary Table S3. List of the 16,450 orthologous gene families that appear on the 53 E. coli strains and their associated gene names, locus tags, gene IDs, protein IDs.
Supplementary Table S4. List of associated gene pairs detected in this study and their gene names, locus tags across different genomes considered.
Supplementary Table S5. Presence / absence table of the genes in the 53 extant and 52 ancestral strains. The ID of an ancestral strain (internal node of the phylogenetic tree) is defined as the IDs of all the extant strains in its sub-clade joined by the symbol ‘+’.
Supplementary Table S6. List of 34 GO terms used to detect phage or mobile element association of genes.
Supplementary Table S7. A summary of bootstrap value, number of genes in the older and younger strains, number of genes gained and lost, and also the number of cogain pairs involved for different branches of the phylogenetic tree.

SUPPLEMENTARY DATA

Supplementary Data S1. Newick format of the 70 strain phylogenetic tree (Supplementary Figure S2).
